# Staff views on overdose prevention in permanent supportive housing

**DOI:** 10.1186/s12954-025-01215-x

**Published:** 2025-04-18

**Authors:** Kelly M. Doran, Allison Torsiglieri, Jocelyn Moran, Stephanie Blaufarb, Annie Y. Liu, Emily Ringrose, Cooper Urban, Lauren Velez, Patricia Hernandez, Megan A. O’Grady, Donna Shelley, Charles M. Cleland

**Affiliations:** 1 Department of Emergency Medicine, NYU Grossman School of Medicine, 227 E. 30th Street, Ground Floor, New York, NY 10016 USA; 2 Department of Population Health, NYU Grossman School of Medicine, New York, USA; 3 Metro Team, Corporation for Supportive Housing, New York, USA; 4 Department of Public Health Sciences, University of Connecticut School of Medicine, Farmington, USA; 5 Department of Public Health Policy and Management, NYU School of Global Public Health, New York, USA

**Keywords:** Overdose, Housing, Homelessness, Harm reduction, Implementation science

## Abstract

**Background:**

Permanent supportive housing (PSH) is the gold standard intervention for chronic homelessness, but PSH tenants face high risk for overdose due to a combination of individual and environmental risk factors. Little research has examined overdose prevention in PSH.

**Methods:**

We conducted baseline surveys with staff from 20 New York PSH buildings participating in an overdose prevention technical assistance intervention study. PSH staff from participating buildings were invited via email to complete a brief online survey about their knowledge of overdose and perspectives on implementing overdose prevention practices in PSH.

**Results:**

Surveys were completed by 178 staff of 286 invitations sent (response rate 62.2%). Average score on the Brief Opioid Overdose Knowledge (BOOK) questionnaire was 8.62 (SD 2.64) out of 12 points. Staff felt very positively (91.6–97.2% agreed or completely agreed) regarding the appropriateness and acceptability of implementing overdose prevention practices in PSH, but less certain about the feasibility of implementing these practices (62.4–65.5% agreed or completely agreed). Most (77.3%) felt it was mostly or definitely true that overdose prevention was a top priority in their building. Most PSH staff (median = 85.0%) but fewer tenants (median = 22.5%) had received a naloxone kit and training in overdose response.

**Conclusion:**

Staff feel positively about the acceptability and appropriateness of implementing overdose response practices in PSH, but somewhat more uncertain about the feasibility of implementing these practices. This study’s results help hone targets for interventions to help PSH buildings take steps to reduce tenant overdose risk.

**Supplementary Information:**

The online version contains supplementary material available at 10.1186/s12954-025-01215-x.

## Introduction

Homelessness and housing instability are strongly associated with increased overdose risk [1]. Overdose is the leading cause of death among people experiencing homelessness across the United States [2, 3]. Permanent supportive housing (PSH) – permanent, subsidized housing paired with voluntary supportive services such as case management – is a key part of national strategic plans to end homelessness. PSH following a Housing First model, whose principles include that housing is provided without prerequisites such as sobriety, is supported by decades of evidence showing that it is highly effective in resolving an individual’s homelessness [4–8]. 

Amidst the clear successes of PSH, evidence regarding its effects on substance use outcomes remains mixed [9]. Most concerningly, individuals remain at high risk for overdose even once housed in PSH. In a survey conducted in 2019–2020, two-thirds of PSH agencies in New York reported at least one opioid-involved overdose among their tenants in the past year [10]. In New York City, 8% of *all* overdose deaths in 2022 occurred in PSH or single-room occupancies, far exceeding the share of the population in such housing [11]. In addition to potential individual-level factors inherent in how PSH is targeted (e.g., to people experiencing chronic homelessness and concurrent health conditions such as substance use disorders and mental illness), the building-level and larger social-structural environment in PSH and other housing settings such as single-room occupancies (SROs) may contribute to overdose risks [12–16]. 

Past research—much of it conducted in Canada using qualitative methods including ethnography and in-depth interviews—has shed light on structural drivers of overdose risk in housing settings. Fleming, et al. (2024) provided a succinct review of the past literature and summary of building-level and larger structural risk factors that may contribute to overdose risk in housing settings [13]. For example, Bardwell, et al.’s work using qualitative interviews, focus groups, and ethnographic observation revealed barriers faced by tenants including the threat of eviction and housing loss if their drug use became known [12]. A more recent ethnographic study of a building providing both shelter and housing services in Vancouver by Scher, et al. similarly identified stigma and fear (of housing loss) as barriers to tenant use of an onsite supervised consumption room [16]. Such factors may contribute to tenants using drugs alone in their apartments without others aware, a strong risk factor for fatal overdose [13, 16]. While not focused specifically on overdose, other studies of PSH have identified additional pertinent social-structural risks including being situated in neighborhoods with high levels of substance use, certain social network characteristics, and contentious relationships with caseworkers [15, 17, 18]. 

Evidence-based harm reduction practices have not been broadly implemented in PSH [10, 13, 19, 20]. There are no federal mandates requiring their implementation, and integration of harm reduction principles and related initiatives in PSH is highly variable [21–24]. Little is known about the barriers to implementation of overdose prevention practices in PSH. Our team is conducting a community-partnered trial to examine the impact of a technical assistance intervention designed to help PSH agencies implement overdose prevention practices [25]. In this paper, we report results of baseline survey questionnaires conducted with PSH staff on their overdose knowledge and feelings about implementing overdose prevention practices in PSH. Particularly amidst a dearth of existing research on this topic, these early findings can help guide harm reduction efforts in PSH.

## Methods

### Study Design

We report results from baseline survey questionnaires conducted with PSH building staff as part of a stepped wedge randomized controlled trial (RCT) of an intervention providing implementation support for overdose prevention in PSH (the PSH Overdose Prevention [POP] Study) [25]. The study was approved by the Institutional Review Board at NYU Grossman School of Medicine. Participation was voluntary. PSH staff were sent an informed consent document for review prior to deciding whether to participate; a waiver of written (i.e., signed) informed consent was received for this study. Participants received a $10 Amazon gift card for completing the survey and were also entered into a raffle for one of two $50 gift cards.

### Study Population

 Survey questionnaires were sent to PSH staff from the 20 buildings participating in the POP Study. All participating buildings are located in New York City (NYC) or New York’s Capital Region. The POP Study protocol, including details about selection of participating buildings, has been previously described [25]. In brief, buildings were selected based on having concerns related to tenant overdose (e.g., based on past overdoses in the building) and to include a diversity of building sizes (range: approximately 16 to 140 PSH tenants), locations (5 buildings in NY’s Capital Region and 15 in NYC [including 9 in the Bronx, 3 in Manhattan, and 3 in Brooklyn]), populations served (including buildings that focus on populations with HIV, mental illness, substance use disorders, and women), and agency staffing and infrastructure. All participating buildings are congregate PSH (i.e., PSH tenants live together, sometimes along with other non-PSH low-income tenants, in one building versus a “scattered site model”) and operated by nonprofit organizations.

Buildings were asked to share the contact information for all staff that interacted with supportive housing tenants in the building, and staff who supervised other staff working directly with tenants. They were specifically instructed to include frontline staff, supervisors, program directors, and agency leaders as well as, to the extent possible, security, maintenance, and overnight and per diem staff. To be eligible for the survey, individuals had to be currently working at a participating PSH building or have worked for one in the past 6 months, and be able to read and respond in English. The survey questionnaire began with a question confirming eligibility.

### Measures

Surveys were administered from October–November 2023. Survey invitations were sent by e-mail; the invitation e-mails contained a unique, secure, clickable weblink for staff to complete the survey. Staff were reminded about the survey through follow-up e-mails and phone calls.

Participants completed the survey questionnaire on the REDCap platform [26]. The questionnaire was designed to be brief (completion time approximately 10–20 min). Responses were confidential; staff were not asked to provide identifying information. Questionnaires asked staff for basic information about their role/job category and demographic information. Staff overdose knowledge was measured using the Brief Opioid Overdose Knowledge (BOOK) questionnaire [27]. We used single-item questions to measure staff opinions on intervention (a) appropriateness, (b) feasibility, and (c) acceptability for three categories of overdose prevention strategies: (1) overdose response practices, (2) harm reduction practices, and (3) practices to support tenants in receiving substance use treatment. Each of these overdose prevention practice categories was described in the survey questionnaire text [full text available in Additional File 1]. These single-item measures were adapted from Weiner, et al.’s Intervention Appropriateness Measure, Feasibility of Intervention Measure, and Acceptability of Intervention Measure, each of which were originally 4 items [28]. We asked about the three categories of overdose response practices rather than the twenty individual practices to minimize response burden. We additionally measured organizational priority for overdose prevention using 7 items adapted from prior research (e.g., “At this building, there is a big push to take steps to prevent tenant overdose”) [29]. The full appropriateness, feasibility, acceptability, and organizational priority items used for this study are provided as an additional file [Additional File 1].

A subset of two staff members from each PSH building (40 participants total) answered additional questions related to overdose (e.g., number of tenant fatal and nonfatal overdoses) and current overdose prevention practices in their building. The staff members completing these additional building self-assessment questions were selected based on their role in the buildings (i.e., a leader or other key staff person who would have knowledge about the building’s overdose prevention practices).

The survey was developed and revised with input from the primary study community partner, Corporation for Supportive Housing, as well as from a Study Advisory Board that includes individuals with lived experience as PSH tenants as well as representatives from relevant community, governmental, and advocacy organizations.

### Analysis

We present descriptive statistics for this brief report [26]. SAS 9.4 was used for analyses. Missing data was minimal and is noted as applicable.

## Results

Survey invitations were sent to 286 staff from 20 PSH buildings; 178 staff members completed the survey (response rate 62.2%). The number of participants per building ranged from 3 to 17 (response rate range 44.4–100% for the different buildings). Participant demographics are reported in Table 1. Approximately one-third (30.3%) of participants reported working in the building less than 1 year, 33.7% 1–3 years, and 36.0% over 3 years. Participants represented a wide range of staff roles (Table 1).

**Table 1:** Participant characteristics (*N* = 178)^1^

	*N* (%)
Gender (*N* = 176)	
Man	50 (28.4)
Woman	120 (68.2)
Non-binary, gender fluid, gender non-conforming	4 (2.3)
Prefer not to answer	2 (1.1)
Race (*N* = 176)	
American Indian / Alaska Native	1 (0.6)
Asian	3 (1.7)
Black or African American	78 (44.3)
Native Hawaiian or Other Pacific Islander	1 (0.6)
White	61 (34.7)
More than One Race	12 (6.8)
None of these	4 (2.3)
Prefer not to answer	16 (9.1)
Ethnicity (*N* = 176)	
Hispanic / Latino(a/x)	44 (25.0)
Not Hispanic / Latino(a/x)	122 (69.3)
Prefer not to answer	10 (5.7)
Age (*N* = 176)	
18–24	6 (3.4)
25–44	84 (47.7)
45–64	73 (41.5)
65+	10 (5.7)
Prefer not to answer	3 (1.7)
Job Role (Current Role in the Building)	
Administrative Assistant or Program Manager	9 (5.1)
Agency Leadership (Executive Director and Vice President titles)	11 (6.2)
Associate/Senior Director, Program Lead, or Department Head	29 (16.3)
Building Director and other Directors (e.g., clinical, programs)	11 (6.2)
Coordinators (e.g., residential, service)	4 (2.2)
Case Manager	46 (25.8)
Case Manager Supervisor	13 (7.3)
Counselor (including overnight)	5 (2.8)
Front Desk / Receptionist	7 (3.9)
Maintenance or Custodial	5 (2.8)
Peer Specialist	10 (5.6)
Security	5 (2.8)
Substance Use Counselor, Harm Reduction Coordinator/Counselor	5 (2.8)
Other Clinical Roles (e.g., mental health provider/counselor, nurse)	9 (5.1)
Other	9 (5.1)
Tenure Working at Building (in any role)	
<6 months	23 (12.9)
6 months to 1 year	31 (17.4)
1 year to 3 years	60 (33.7)
More than 3 years	64 (36.0)

*1. N = 178 unless otherwise noted*

Average score on the Brief Opioid Overdose Knowledge (BOOK) questionnaire was 8.62 (SD 2.64) out of a maximum score of 12 (Table 2). Few staff (12.1%) answered all 12 questions correctly. Mean scores were similar for the three BOOK subcategories: opioid knowledge, overdose knowledge, and overdose response knowledge. The questions most commonly answered correctly were “Narcan (naloxone) will reverse the effect of an opioid overdose” (96.0% of staff gave the correct response) and “Heroin, OxyContin, and fentanyl are all examples of opioids” (89.2% correct). The questions least commonly answered correctly were “If you see a person overdosing on opioids, you can begin rescue breathing until a health worker arrives” (57.1% correct); “Long acting opioids are used to treat chronic ‘round the clock’ pain” (59.1% correct); “Methadone is a long-acting opioid” (62.9% correct); and “All overdoses are fatal (deadly)” (63.1% correctly answered false).

**Table 2:** Staff Brief Opioid Overdose Knowledge (BOOK) questionnaire scores

Total BOOK Score (Range 0–12)	*N*^1^ (%)
0	2 (1.1)
1	2 (1.1)
2	2 (1.1)
3	1 (0.6)
4	10 (5.7)
5	5 (2.9)
6	8 (4.6)
7	17 (9.8)
8	24 (13.8)
9	27 (15.5)
10	33 (19.0)
11	22 (12.6)
12	21 (12.1)
**Mean BOOK Total (Range 0–12) and Subset (Each Range 0–4) Scores**	
Total BOOK Score, Mean (SD)	8.62 (2.64)
Opioid Knowledge Subset Score, Mean (SD)	2.87 (1.16)
Overdose Knowledge Subset Score, Mean (SD)	2.82 (1.25)
Overdose Response Knowledge Subset Score, Mean (SD)	2.92 (1.07)

1. Due to missing responses for some questions, for total scores *N* = 174, for opioid and overdose response knowledge subset scores *N* = 175, for overdose knowledge subset score *N* = 176.

Table 3 shows staff views on the perceived appropriateness, feasibility, and acceptability of implementing three categories of overdose prevention practices in PSH. In general, staff felt very positively regarding the *appropriateness* and *acceptability* of implementing these practices, but less certain about the *feasibility* of implementing them; this trend was consistent for all three overdose prevention practice categories. Table 3 also shows staff agreement with 7 statements related to their perceptions of organizational priority for implementing overdose prevention practices. Most (77.3%) reported that it was “mostly” or “definitely” true that overdose prevention is a top priority in the building where they work. Nearly all reported that staff cared about tenant overdose. However, 41.2% noted that overdose prevention sometimes took “a back seat to other priorities.”

**Table 3:** Staff views on the appropriateness, feasibility, acceptability, and priority of implementing overdose prevention practices in permanent supportive housing (*n* = 178 unless otherwise noted)

	Completely Disagree *n* (%)	Disagree *n* (%)	Neither Agree nor Disagree *n* (%)	Agree *n* (%)	Completely Agree *n* (%)
**Intervention Appropriateness** (“X seem like a good match for this building”)					
Overdose response practices^1^	2 (1.1)	3 (1.7)	9 (5.1)	58 (32.6)	106 (59.6)
Practices related to harm reduction for substance use	2 (1.1)	2 (1.1)	11 (6.2)	65 (36.5)	98 (55.1)
Practices to support tenants in receiving substance use treatment (*n* = 177)	3 (1.7)	0	5 (2.8)	64 (36.2)	105 (59.3)
**Intervention Feasibility** (“X seem easy to implement in this building”)					
Overdose response practices	1 (0.6)	15 (8.4)	51 (28.7)	80 (44.9)	31 (17.4)
Practices related to harm reduction for substance use	1 (0.6)	11 (6.2)	54 (30.3)	78 (43.8)	34 (19.1)
Practices to support tenants in receiving substance use treatment (*n* = 177)	1 (0.6)	15 (8.5)	45 (25.4)	75 (42.4)	41 (23.2)
**Intervention Acceptability** (“I welcome X in this building”)					
Overdose response practices	2 (1.1)	0	3 (1.7)	57 (32.0)	116 (65.2)
Practices related to harm reduction for substance use	2 (1.1)	1 (0.6)	7 (4.0)	56 (31.6)	111 (62.7)
Practices to support tenants in receiving substance use treatment (*n* = 176)	2 (1.1)	0	4 (2.3)	55 (31.3)	115 (65.3)
**Organizational Priority** (*n* = 177 unless noted otherwise)	**Not True** n (%)	**Slightly True** n (%)	**Somewhat True** n (%)	**Mostly True** n (%)	**Definitely True** n (%)
Overdose prevention is a top priority in this building. (*n* = 176)	5 (2.8)	10 (5.7)	25 (14.2)	50 (28.4)	86 (48.9)
In this building, overdose prevention takes a back seat to other priorities.	104 (58.8)	21 (11.9)	27 (15.3)	18 (10.2)	7 (4.0)
Staff at this building put a lot of effort into trying to prevent tenant overdose.	5 (2.8)	16 (9.0)	32 (18.1)	37 (20.9)	87 (49.2)
Staff in this building think that implementation of strategies to prevent tenant overdose is important.	1 (0.6)	5 (2.8)	12 (6.8)	39 (22.0)	120 (67.8)
One of this building’s goals is to integrate best practices for OD prevention.	1 (0.6)	8 (4.5)	24 (13.6)	48 (27.1)	96 (54.2)
Staff here don’t care about tenant overdose prevention.	164 (92.7)	4 (2.3)	3 (1.7)	4 (2.3)	2 (1.1)
At this building, there is a big push to take steps to prevent tenant overdose.	7 (4.0)	14 (7.9)	32 (18.1)	39 (22.0)	85 (48.0)

1. Practice categories are described in Additional File 1.

Two leaders from each building (*n* = 40) additionally completed self-assessment surveys related to overdoses in their building and their building’s current overdose prevention practices. A majority (*n* = 24, 60.0%) reported that there had been no *fatal* overdoses amongst their building’s tenants in the past 6 months; 6 (15.0%), 3 (7.5%), 3 (7.5%), 3 (7.5%), and 1 (2.5%) reported 1, 2, 3, 4, and 5 *fatal* overdoses in the past 6 months, respectively. Approximately half (*n* = 22, 55.0%) reported no *non-fatal* tenant overdoses in the past 6 months, while 6 (15.0%) reported 1 *non-fatal* overdose in the past 6 months and 12 (30.0%) reported 2 or more *non-fatal* overdoses in the past 6 months (range 2–20).

Regarding percentage of supportive housing *staff* reported by building leaders to have received a naloxone kit and training in naloxone use and overdose response, 2 leaders (5.0%) reported that none of their staff had received such training and 18 (45.0%) reported that 100% of their staff had received it, with a range of responses in between (median = 85.0%, IQR = 22.5–100%). Regarding percentage of *tenants* reported by building leaders to have received a naloxone kit and training in naloxone use and overdose response, 4 leaders (10.0%) reported that none of their tenants had received such training and 3 (7.5%) reported that 100% of their tenants had received it, with a wide range of responses in between (median = 22.5%, IQR = 10.0–50.0%). In general, exact concordance in responses between the two leaders surveyed from the same building was low.

## Discussion

Findings from this survey provide actionable information that can help guide efforts to improve overdose prevention in PSH. First, staff generally found the idea of implementing overdose prevention practices in PSH to be highly acceptable and appropriate, spanning all categories of overdose prevention practice types. Responses indicated that staff cared about overdose prevention and that there was generally a high organizational priority for overdose prevention. These positive staff feelings are strengths that could be capitalized on as potential facilitators of overdose prevention implementation efforts. Notably, though past studies have found variability in PSH orientation toward harm reduction [22], our survey results indicated that PSH staff seemed to feel just as positively toward harm reduction-focused overdose prevention practices as they did toward more treatment-focused practices.

Survey findings revealed that potential barriers to overdose prevention practice implementation in PSH may be less related to staff buy-in and more related to practicality and feasibility. Survey results for questions related to overdose prevention practice feasibility were less positive than those for acceptability and appropriateness. Some staff also reported that overdose prevention sometimes took a backseat to other organizational priorities. These findings suggest that implementation strategies should focus on addressing organizational and building level factors to address feasibility and leadership buy-in and prioritization, rather than efforts solely focused on changing staff attitudes toward overdose prevention. Findings related to feasibility and organizational priority are not surprising considering that nonprofit PSH service providers often operate within significant resource constraints. Advocates have noted that budgets for PSH services have not kept up with the needs, and that staff pay is too low. Low staff pay combined with high levels of burnout leads to high staff turnover, which was evident in our survey finding that 30.3% of staff respondents had been with their current organization for one year or less. High staff turnover presents challenges to ensuring that all staff have the proper education and training related to overdose prevention and response, let alone creating a shared understanding or culture grounded in harm reduction. These findings suggest that larger policy changes – such as ensuring adequate payment for PSH services and robust staffing – may be necessary for PSH agencies to most effectively address tenant overdose.

This study identified concrete gaps related to overdose prevention in PSH that could be addressed with pragmatic action. First, when we asked two staff leaders in the same building to report the number of tenant overdoses in their building, over the same period of time, their responses were rarely the same. This finding suggests a need for uniform monitoring and tracking – and standardized debriefing – related to tenant overdose in PSH. In the aforementioned RCT (the POP Study), PSH buildings receive guidance on tracking important elements related to tenant overdoses (e.g., setting of an overdose, whether naloxone was administered) in a standardized way using whatever secure data system works best for the building, to provide actionable information to improve their overdose prevention and response efforts. Second, specific knowledge gaps related to overdose response were identified; these gaps could be addressed through enhanced staff training on overdose. The importance of regularly updated training is further underscored by emerging changes in the unregulated drug supply that may impact overdose (e.g., increasing xylazine contamination). While still untested, it is possible that enhanced staff training may also increase feelings of staff competence related to overdose, reduce feelings of burnout, and improve retention. Last, we identified gaps in overdose education and naloxone distribution for PSH tenants. If all PSH tenants in a building were trained in responding to overdose and had their own naloxone kits, it could increase the likelihood of a quick and potentially life-saving response to tenant overdose in the building. One promising model is the Tenant Overdose Response Organizers (TORO) program, a tenant-led naloxone training and distribution intervention in single-room occupancies (SROs) in Vancouver, Canada [12]. 

Our findings from PSH in New York echo several findings from research conducted elsewhere. For example, in the study of the TORO program mentioned above, researchers generally found that acceptability of the program was enhanced given the clear need, but feasibility challenges remained such as mixed support of building management and environmental conditions in SROs themselves [12]. In another study, Olding, et al. examined implementation of the “SRO Project,” a pilot project that trained tenants to provide overdose education and naloxone distribution in San Francisco SROs [30]. Qualitative interviews and ethnographic field work conducted in two permanent supportive housing SROs that implemented the SRO Project revealed—similar to findings in our current study—frequent staff turnover, which posed implementation barriers including loss of “institutional knowledge,” confusion about responsibility for the pilot, and negative impacts on tenant trust [30]. A recent study using in-depth qualitative interviews with PSH service providers across Canada examined barriers and facilitators to addressing high-risk tenant behaviors, including but not limited to overdose [15]. This study also revealed frequent staff turnover, along with challenges including organizational resource limitations, staff training and supervision, and a lack of needed community resources [15]. Notably, the study revealed some variation in barriers and facilitators depending on factors such as program organization and resources in the local environment, highlighting the potential need for tailored interventions [15]. 

Urgent action is needed to reduce the risks for fatal overdose faced by tenants in PSH, as well as in shelters and other settings serving people experiencing homelessness. In a recent commentary, Fleming and colleagues describe this need and provide recommendations to guide such action [13]. They highlight the importance of addressing larger contextual factors (e.g., the housing and drug policy environment) as well as intervening at the more local housing environment, and they emphasize that interventions should be guided by people who use drugs and bring lived expertise [13]. Overall, there remains limited evidence about how to most effectively implement evidence-based overdose prevention practices in housing settings like PSH, SROs, and shelters. The previously mentioned pilot projects in San Francisco [30] and Vancouver [12, 16] provide promising ideas for overdose prevention interventions in housing environments. Guidance on overdose prevention in hotel settings used for people experiencing homelessness during the COVID-19 pandemic may also be relevant for PSH and other settings [31, 32]. In New York, our research team is conducting a study examining the impact of technical assistance to help PSH buildings implement improved overdose prevention practices [25]. The overdose prevention practices and technical assistance activities—which include a written toolkit and training videos, tailored practice facilitation sessions for each building, and group learning collaboratives—address several of the needs described in the current study [25]. 

Our findings may not be fully transferable to settings outside New York. Additionally, the PSH buildings from which staff were surveyed do not represent a random selection of PSH buildings in New York. Buildings selected for this study do, however, represent a diverse group of buildings (e.g., in size, tenant subpopulations, and geography) that had demonstrated need related to tenant overdose. Additionally, there may have been bias in which staff completed surveys (e.g., staff with particularly positive or particularly negative views related to overdose). For those staff who did complete the survey, there may have been social desirability bias in the responses, though we attempted to limit this by ensuring confidentiality and specifying that responses would only be seen by academic researchers and not by leaders or other staff from their agencies. Despite these limitations, we believe our study is an important contribution to the literature as the first study to examine PSH staff knowledge and attitudes related to overdose prevention practice implementation in PSH.

## Conclusion

In this survey of PSH staff in New York, we identified both assets and gaps related to overdose prevention. Staff felt positively about the acceptability and appropriateness of implementing overdose response practices in PSH, but were more uncertain about the feasibility of implementing these practices. These findings can be used to help hone targets for interventions to reduce tenant overdose risk in PSH.

## Electronic supplementary material

Below is the link to the electronic supplementary material.

Supplementary Material 1

## Data Availability

The full study protocol has been previously published. Copies of study questionnaires are available by request to the corresponding author. Deidentified survey data may be made available in aggregate to qualified researchers who have a research question appropriate to the data and of potential benefit to PSH tenants, with approval of the Study Advisory Board and after completion of a data use agreement. Requests should be made by e-mail to the corresponding author.

## Abbreviations

BOOK: Brief Opioid Overdose Knowledge (questionnaire)
POP Study: Permanent Supportive Housing Overdose Prevention Study
PSH: Permanent supportive housing
RCT: Randomized controlled trial
SD: Standard deviation
