# Supplementary material for: Staff views on overdose prevention in permanent supportive housing
Source: Harm Reduct J. 2025 Apr 18;22:59. doi: 10.1186/s12954-025-01215-x (PMC12007226; doi:10.1186/s12954-025-01215-x)
Supplement: Supplementary file 1 — Supplementary Material 1 [file 12954_2025_1215_MOESM1_ESM.docx]

**Additional File 1 – Intervention Appropriateness, Feasibility, and Acceptability Questions**

We created single-item questions to measure staff opinions on intervention a) appropriateness, b) feasibility, and c) acceptability for three categories of overdose prevention strategies: 1) overdose response practices, 2) harm reduction practices, and 3) practices to support tenants in receiving substance use treatment. Each overdose prevention practice category was described in the survey questionnaire text. The single-item measures were adapted from Weiner, et al.’s 3-item Intervention Appropriateness Measure (IAM), Feasibility of Intervention Measure (FIM), and Acceptability of Intervention Measure (AIM).

| *Please read the following instructions carefully:* The next set of questions asks your opinion on ***overdose response practices*** in the building. Such practices include: having a written overdose prevention and response plan; holding debriefings and providing emotional support for tenants and staff after overdoses; making naloxone (Narcan) kits readily available throughout the building; offering overdose response trainings and naloxone (Narcan) kits to tenants and staff; and systematic tracking of overdoses in the building. If you are completing this survey on your phone, turn your phone sideways for better viewing. |
| --- |

|  | **Completely disagree** | **Disagree** | **Neither agree nor disagree** | **Agree** | **Completely**  **agree** |
| --- | --- | --- | --- | --- | --- |
| **IAM1**. **Overdose response practices** seem like a good match for this building. | ➀ | ➁ | ➂ | ➃ | ➄ |
| **FIM1**. **Overdose response practices** seem easy to implement in this building. | ➀ | ➁ | ➂ | ➃ | ➄ |
| **AIM1**. I welcome **overdose response practices** in this building. | ➀ | ➁ | ➂ | ➃ | ➄ |

| *Please read the following instructions carefully:* The next set of questions asks your opinion on practices in this building related to ***harm reduction for substance use***. Such practices for supportive housing tenants include: supporting a harm reduction model and not penalizing tenant drug use; taking steps to minimize tenants using drugs alone; educating staff and tenants on harm reduction and current risks related to the drug supply; having discussions with all supportive housing tenants about safely managing drug use and decreasing their overdose risk; holding events for staff and tenants to reduce stigma toward people who use drugs; requiring trauma-informed care training for staff; and providing extra support for tenants in high-risk periods for overdose such as in transition periods. If you are completing this survey on your phone, turn your phone sideways for better viewing. |
| --- |

| ***Please note:*** *these questions will appear similar to the set of questions you just completed, but for this set of questions we are asking about practices related to harm reduction.* | **Completely disagree** | **Disagree** | **Neither agree nor disagree** | **Agree** | **Completely**  **agree** |
| --- | --- | --- | --- | --- | --- |
| **IAM2**. Practices related to **harm reduction for substance use** seem like a good match for this building. | ➀ | ➁ | ➂ | ➃ | ➄ |
| **FIM2**. Practices related to **harm reduction for substance use** seem easy to implement in this building. | ➀ | ➁ | ➂ | ➃ | ➄ |
| **AIM2**. I welcome practices in this building related to **harm reduction for substance use**. | ➀ | ➁ | ➂ | ➃ | ➄ |

| *Please read the following instructions carefully:* The next set of questions asks your opinion on practices to ***support building tenants in receiving substance use treatment***. Such practices for supportive housing tenants include: educating staff and tenants on different substance use disorder (SUD) treatment types; regularly assessing tenant substance use and SUD treatment needs and interest; establishing referral pathways to local MOUD (medications to treat opioid use disorder, like methadone and Suboxone) providers; providing tenants with access to professional peer support models related to substance use; helping tenants access SUD treatment (for example, provide transportation); and providing programming and resources for tenants in SUD treatment, who are trying to reduce use, or who are maintaining sobriety. If you are completing this survey on your phone, turn your phone sideways for better viewing. |
| --- |

| ***Please note:*** *these questions will appear similar to the sets of questions you just completed, but in this set of questions we are asking about practices to support tenants in receiving substance use treatment.* | **Completely disagree** | **Disagree** | **Neither agree nor disagree** | **Agree** | **Completely**  **agree** |
| --- | --- | --- | --- | --- | --- |
| **IAM3**. Practices to **support tenants in receiving substance use treatment** seem like a good match for this building. | ➀ | ➁ | ➂ | ➃ | ➄ |
| **FIM3**. Practices to **support building tenants in receiving substance use treatment** seem easy to implement. | ➀ | ➁ | ➂ | ➃ | ➄ |
| **AIM3**. I welcome practices to **support building tenants in receiving substance use treatment**. | ➀ | ➁ | ➂ | ➃ | ➄ |

| **Please provide your opinion on how overdose prevention is viewed in your building.** **For each of the items below please answer "not true," "slightly true," "somewhat true," "mostly true," or "definitely true". If you are completing this survey on your phone, turn your phone sideways for better viewing.** | **Not true** | **Slightly true** | **Somewhat true** | **Mostly true** | **Definitely true** |
| --- | --- | --- | --- | --- | --- |
| **OP1**. Overdose prevention is a top priority in this building. | ➀ | ➁ | ➂ | ➃ | ➄ |
| **OP2**. In this building, overdose prevention takes a back seat to other priorities. | ➀ | ➁ | ➂ | ➃ | ➄ |
| **OP3**. Staff at this building put a lot of effort into trying to prevent tenant overdose. | ➀ | ➁ | ➂ | ➃ | ➄ |
| **OP4**. Staff in this building think that implementation of strategies to prevent tenant overdose is important. | ➀ | ➁ | ➂ | ➃ | ➄ |
| **OP5**. One of this building’s goals is to integrate best practices for overdose prevention. | ➀ | ➁ | ➂ | ➃ | ➄ |
| **OP6**. Staff here don’t care about tenant overdose prevention. | ➀ | ➁ | ➂ | ➃ | ➄ |
| **OP7**. At this building, there is a big push to take steps to prevent tenant overdose. | ➀ | ➁ | ➂ | ➃ | ➄ |
